# Therapeutic Approaches for Chronic Obstructive Pulmonary Disease (COPD) Exacerbations

**DOI:** 10.3390/pathogens11121513

**Published:** 2022-12-10

**Authors:** Yehudis Rosenwasser, Irene Berger, Zvi G. Loewy

**Affiliations:** 1 College of Pharmacy, Touro University, 230 West 125th Street, New York, NY 10027, USA; 2 School of Medicine, New York Medical College, Valhalla, NY 10595, USA

**Keywords:** oral disease, respiratory disease, microbiome, GOLD guidelines

## Abstract

Chronic Obstructive Pulmonary Disease (COPD) is a progressive pulmonary disorder underpinned by poorly reversible airflow resulting from chronic bronchitis or emphysema. The prevalence and mortality of COPD continue to increase. Pharmacotherapy for patients with COPD has included antibiotics, bronchodilators, and anti-inflammatory corticosteroids (but with little success). Oral diseases have long been established as clinical risk factors for developing respiratory diseases. The establishment of a very similar microbiome in the mouth and the lung confirms the oral-lung connection. The aspiration of pathogenic microbes from the oral cavity has been implicated in several respiratory diseases, including pneumonia and chronic obstructive pulmonary disease (COPD). This review focuses on current and future pharmacotherapeutic approaches for COPD exacerbation including antimicrobials, mucoregulators, the use of bronchodilators and anti-inflammatory drugs, modifying epigenetic marks, and modulating dysbiosis of the microbiome.

## 1. Introduction

Chronic Obstructive Pulmonary Disease (COPD) is the third leading cause of death worldwide. COPD can be divided into two clinical phenotypes: emphysema and chronic bronchitis. Pathologically, emphysema is correlated with the enlargement of distal air spaces. Chronic bronchitis is defined clinically as a productive cough that lasts at least three months, recurring for a minimum of two consecutive years. Many factors contribute to the development of COPD, including genetic factors (alpha1-antitrypsin deficiency), pollution, cigarette smoke, and occupational exposure to various chemicals.

COPD manifests as an inflammatory condition involving the airways, lung parenchyma, and pulmonary vasculature. Inhalation exposure can trigger an inflammatory response. The inflammatory response results in a decrease in the forced expiratory volume, and tissue destruction leads to airflow limitation. The inability to fully exhale promotes an elevation in carbon dioxide levels. Airflow limitation is the main pathophysiologic feature of COPD.

COPD exacerbation correlates with bacterial colonization of the upper and lower airways. In addition to bacteria colonization, exacerbations also correspond to a rise in acute respiratory viral infections including influenza, rhinovirus, and coronavirus. Collectively, bacterial and viral pathogens introduce new antigens that result in the secretion of chemokines leading to the destruction of the bronchial epithelium and resulting in clinical exacerbation.

Previously, we demonstrated that a correlation exists between poor oral health and the incidence of COPD [1,2]. Although the oral cavity and the lung are perceived as different clinical systems, they are indeed part of a continuum predicated upon their microbiomes. COPD severity can be exacerbated by exposure to pathogenic microorganisms including *Pseudomonas aeruginosa*, *Moraxella catarrhalis*, *Haemophilus influenzae*, and *Streptococcus pneumoniae.* The pathogens implicated in COPD pose a formidable therapeutic challenge since they are embedded within biofilms [3], and biofilms have proven to be difficult to control and eradicate [4].

COPD exacerbations are classified as mild, moderate, or severe. Treatments for exacerbations differ based on their classification. Outpatients experiencing mild exacerbations are managed with short-acting bronchodilators only. Outpatients experiencing moderate exacerbations are generally treated with short-acting bronchodilators and antibiotics, with or without oral corticosteroids, depending on clinical signs and symptoms. Patients experiencing severe exacerbations are hospitalized with possible intensive care admission [5]. There are many potential causes of COPD exacerbations and many pharmacological options for managing COPD exacerbations. Pharmacological therapies target both the symptoms and the cause of the exacerbation.

The Global Initiative for Chronic Obstructive Pulmonary Disease (GOLD) publishes guidelines that are widely accepted by clinicians in the United States and internationally as the standard on which to base therapy. These guidelines offer recommendations on how to initiate therapy in newly diagnosed COPD patients, and they offer recommendations on how to modify therapy in patients who experience frequent exacerbations. In newly diagnosed COPD patients, the GOLD guidelines recommend initiating therapy based on the severity of the illness. In patients who experience frequent exacerbations, the guidelines recommend modifying the therapy based on the medications that the patient takes. Recommendations are offered based on eosinophil count, smoking history, and the presence of bronchitis. In patients whose exacerbations are not adequately treated with guideline-recommended therapy, investigative and alternative treatments can be considered. The GOLD guidelines also offer recommendations on how to treat acute exacerbations, including antimicrobials, mucoregulators, the use of bronchodilators, and anti-inflammatories [5]. Each of these drug classes will be further explored with an in-depth analysis of the published literature as well as a look at the future describing novel technologies predicated on epigenetics, analyzing their place in therapy for treating acute COPD exacerbations.

## 2. Materials and Methods

A meta-analysis of previously published studies was conducted. The descriptive data were collected. The search terms used included COPD exacerbation, anti-microbials, bronchodilators, anti-inflammatories, genomics, microbial dysbiosis, etc. MEDLINE, EMBASE, related internet websites, and reference lists were searched from 1997 to 2022 to identify appropriate papers that addressed the objectives of this review. Publications were reviewed independently by three investigators. The investigators extracted the data and inspected each reference identified during the search and applied the inclusion criteria. In cases where the same studies were reported in more than one publication, the study’s results were accounted for only once. The electronic search was followed by extensive hand searching using reference lists from the identified articles. Publications written in English were reviewed exclusively. The search method used was designed to strengthen existing concepts and to identify therapeutic candidates for upcoming research studies.

## 3. COPD Exacerbations and Current Treatment Results

### 3.1. Therapeutic Approaches

#### 3.1.1. Antimicrobials

##### Antibiotics

One study evaluating the causes of COPD exacerbations in hospitalized patients found that 78% of cases resulted from bacterial or viral infections (54.7% of the exacerbations were related to bacterial infections, and 48.4% of the exacerbations were related to viral infectious triggers) [6]. These findings indicate that respiratory infections are strongly associated with most cases of COPD exacerbations. Despite this, the use of antibiotics in the treatment of COPD exacerbations remains controversial. When warranted, the use of antibiotics results in positive patient outcomes, regardless of which antibiotic was utilized. In a systematic review that included 10 trials and a total of 917 patients, the effects of antibiotic therapy in treating moderate-to-severe COPD exacerbations were evaluated. The results demonstrated a 77% reduction in mortality rates, a 44% reduction in sputum purulence, and a 53% reduction in treatment failure in patients included in stratification [7]. However, antibiotics are not recommended for all patients with COPD exacerbations and avoiding overexposure to antibiotics is crucial as it can result in the development of antimicrobial resistance, adverse effects, and other complications. Therefore, when treating COPD exacerbations, it is important to determine which patients will benefit from antibiotic therapy and which will not. In clinical practice, antibiotics are only recommended if there is an increased probability that the patients’ exacerbations are due to bacterial infections. This is determined based on the presence of clinical symptoms of infection. The GOLD guidelines recommend initiating antibiotic therapy in patients with increased sputum purulence accompanied by increased dyspnea, increased sputum volume, or both. Antibiotic therapy is also initiated in patients who require mechanical ventilation. If the patient does not meet this criterion, the patient will not undergo antibacterial therapy for the management of COPD exacerbation, and hence antibiotics should not be administered [5]. Antibacterial treatment is guided by the most commonly detectable pathogens involved in COPD exacerbations. In a meta-analysis including 14 clinical trials, the bacteriology of sputum samples obtained from patients experiencing acute exacerbations was reviewed. The results revealed that the most common pathogens in these patients included both Gram-negative and Gram-positive bacteria. *Haemophilus influenzae*, *Moraxella catarrhalis*, and *Streptococcus pneumoniae* were the most prevalent bacteria, followed by the *Haemophilus parainfluenzae*, *Staphylococcus aureus*, *Pseudomonas aeruginosa*, and *Enterobacteriaceae* species [8]. In outpatients, the recommended treatment involves an aminopenicillin with clavulanic acid, a macrolide, or a tetracycline [5]. In hospitalized patients, sputum cultures should be obtained prior to initiating empirical antibacterial therapy. In hospitalized patients without risk factors for *Pseudomonas aeruginosa*, empiric antibiotic treatment usually involves a respiratory fluoroquinolone (such as levofloxacin or moxifloxacin) or a third-generation cephalosporin, such as ceftriaxone or cefotaxime. In hospitalized patients who present risk factors for *Pseudomonas aeruginosa*, the agents of choice include cefepime, ceftazidime, or piperacillin-tazobactam [5]. One retrospective analysis examined patients treated in the intensive care setting for severe, infectious COPD exacerbations. The results revealed that empiric use of antipseudomonal antibiotics did not improve patient outcomes in those treated in the ICU setting for severe infectious COPD exacerbations. This study was warranted because while undertreatment is associated with increased rates of morbidity and mortality, overtreatment can harm patients and may promote the colonization of *Pseudomonas aeruginosa* and other multi-drug-resistant strains of bacteria. The results of this study demonstrated that there was no statistically significant difference in 30-day mortality in the ICU between those that received antipseudomonal coverage versus those that did not after adjusting for age, gender, illness severity, and comorbidities [9]. Based on these findings, it is reasonable to reserve the use of antipseudomonal antibiotics for hospitalized patients with risk factors for *Pseudomonas aeruginosa* infection. If the cultures are obtained prior to initiating empiric therapy, after the culture results return, antibiotics should be de-escalated appropriately [10].

The updated GOLD guidelines recommend using antibiotics for a duration of five to seven days [5]. A meta-analysis further analyzed the appropriate duration of antibiotic therapy. The authors found that there was no significant difference in clinical improvement in patients who received short-course antibiotics (five days or fewer) compared with those who received antibiotics for the conventional treatment duration of seven to ten days. The results demonstrate that short courses of antibiotics for the treatment of mild-to-moderate exacerbations of COPD are as effective as conventional treatment durations of seven to ten days. A shorter duration of therapy is beneficial because it minimizes the adverse effects associated with antimicrobial treatment and improves compliance [11].

While the GOLD guidelines list several antibiotic agents that may be considered as options for the treatment of exacerbations, the guidelines do not offer recommendations regarding which specific agent is superior. Several studies were conducted to determine the impact of the choice of antibiotics used to treat adults with COPD exacerbations. Placebo-controlled trials demonstrated benefits in survival, length of hospital stay, and time until exacerbation when incorporating antibiotics in the treatment of exacerbations that resulted from infectious triggers. Several comparison trials were later conducted to determine the optimal choice of empiric therapy. In the randomized, controlled GLOBE trial, the effects of a five-day course of gemifloxacin (a fluoroquinolone) were compared to a seven-day course of clarithromycin (a macrolide). The results showed no significant difference in the signs and symptoms of acute exacerbation, but a significant difference was seen in the eradication of the causative respiratory pathogen. The gemifloxacin group had an 86.7% bacteriologic success rate compared to 73.1% in the clarithromycin group. Furthermore, patients that received a five-day course of gemifloxacin experienced significantly lower rates of recurrent exacerbations after 26 weeks compared to those that received a seven-day course of clarithromycin [12]. In the landmark MOSAIC trial, patients were randomized to either moxifloxacin (a fluoroquinolone) or standard therapy with amoxicillin, cefuroxime, or clarithromycin. The study found a significant improvement in the symptoms of those who completed therapy with moxifloxacin compared to those who received standard therapy. Additionally, there was a superior bacteriologic response of 91.5% in the moxifloxacin treatment arm compared to 81% in the standard therapy arm. The trial also demonstrated the long-term benefits of therapy with moxifloxacin. At five months follow-up, the time until the next exacerbation was prolonged in those that were treated with moxifloxacin compared to those that received the standard therapy (131 days until the next exacerbation in the moxifloxacin group compared to 104 days in the standard therapy group) [13]. The results of these two randomized controlled trials suggest that fluoroquinolones demonstrate superiority with regard to efficacy in the treatment of COPD exacerbations. Statistically significant differences in the outcomes were not seen at seven to fourteen days; however, there were significant benefits seen in cure rates, time until next exacerbation, and the need for additional antibiotics at follow-up beyond the first seven to fourteen days after the initiation of therapy. While these results favor fluoroquinolones, it is important to use risk stratification markers and reserve the use of these agents for patients at risk of poor outcomes to prevent acquired antibiotic resistance with this valuable class of antibiotics [14]. A retrospective, cohort analysis was performed to analyze the difference in outcomes in patients older than 65 suffering from acute exacerbations of COPD. The study compared the use of broad-spectrum antibiotics versus narrow-spectrum agents in this patient population. The authors note that they focused on patients in this age group because old age is a risk factor for exacerbations, and this group represents a large portion of those that are hospitalized for COPD exacerbations. In the study, broad-spectrum antibiotics consisted of fluoroquinolones, antipseudomonal penicillins, cephalosporins, or a combination of aminopenicillin and beta-lactamase inhibitors, with the majority of patients in this group receiving fluoroquinolones. Narrow-spectrum agents included sulfamethoxazole/trimethoprim, azithromycin, doxycycline, or aminopenicillins, with the majority of patients receiving azithromycin. The results demonstrated that there was no difference in outcomes between the two treatment arms, including transfer to the ICU 48 h post-admission, requiring mechanical ventilation 48 h post-admission, 30-day hospital readmission due to COPD exacerbations, and increased dyspnea. These results contradict the results seen in the MOSAIC and GLOBE trials and may be due to numerous limitations within this study. One major limitation was the high rate of azithromycin use in the narrow-spectrum group; azithromycin use is commonly limited since there are often increased rates of resistance to macrolides contributing to treatment failure. This is a possible explanation for the contradictory results from this trial compared to the two other landmark trials [15]. In a similarly designed retrospective, cohort study, hospitalized patients that received treatment with azithromycin for COPD exacerbations were compared to patients receiving beta-lactams. The specific beta-lactams included in the study were ceftriaxone, cefuroxime, cefepime, cefazolin, cephalexin, amoxicillin-clavulanic acid, ampicillin-sulbactam, and piperacillin-tazobactam. The results demonstrated that treatment with azithromycin resulted in lower rates of treatment failure, demonstrated by lower rates of hospital readmission, and a reduction in new antibiotic use during admission [16]. While the results of current studies favor the use of macrolides and fluoroquinolones over beta-lactams, more comparative, randomized trials are warranted at this time. Factors to consider include the risk of multi-drug-resistant organism colonization (such as *Pseudomonas aeruginosa*), resistance rates in relevant regions, and the risk of acquired resistance with the overuse of fluoroquinolones.

Due to the results of the MOSAIC and GLOBE studies, practitioners may be attracted to the use of respiratory fluoroquinolones in all patients seen with moderate-to-severe COPD exacerbations. However, the widespread use of these agents may lead to the emergence of antibiotic resistance and therefore should be reserved for patients that are most likely to benefit from their use [14]. Fluoroquinolones work by binding to and inhibiting the activity of topoisomerase IV and DNA gyrase, the enzymes involved in DNA replication. More specifically, fluoroquinolones bind to the *gyrA* or *gyrB* genes, which are responsible for encoding DNA gyrase as well as the *parC* or *parE* genes, which are responsible for encoding topoisomerase IV. When a fluoroquinolone binds to a complex consisting of the pathogenic organism’s DNA and DNA gyrase or topoisomerase IV, the fluoroquinolone inhibits DNA transcription, translation, and repair and ultimately contributes to cell death [17].

The key pathogens implicated in COPD exacerbations develop fluoroquinolone resistance through chromosomal mutations in the enzymes targeted by the antibiotic. Studies have demonstrated that resistance may be conferred by mutations in *parC* and *parE*, the subunits of topoisomerase IV, and *gyrA* and *gyrB*, the subunits of DNA gyrase. Several reports have attributed the emerging resistance of *Streptococcus pneumoniae* to mutations in *gyrA* or *parC* genes primarily. In a global study which analyzed mutations which contributed to resistance to fluoroquinolones, results demonstrated that the most common mutations included Ser-81 Phe or Tyr and Ser-79 Tyr in *gyrA* and *parC* subunits. *Streptococcus pneumoniae* also demonstrated resistance via efflux of the antibiotics. However, the fluoroquinolones which have a larger bulk at the C-7 position, such as moxifloxacin, are less likely to be impacted by this efflux mechanism [18]. Resistance to fluoroquinolones in Gram-positive organisms, such as *Streptococcus pneumoniae*, is most often seen when there are at least two mutations in the relevant genes (i.e., *gyrA* and *parC*) which minimize the binding of the antibacterial agent to the target site of the pathogen. When only one mutation is present, the resistance is usually weak and has a minimal effect. On the other hand, when two or more mutations are present, the result is greater pathogen resistance [19]. An in vitro study compared the effects of levofloxacin to moxifloxacin. The results demonstrated that strains with more than one mutation are more likely to display resistance to levofloxacin but are likely to remain susceptible to the newer respiratory fluoroquinolones [20]. The structures of quinolones were eventually modified to help protect against mechanisms of resistance and improve coverage against respiratory pathogens such as *Streptococcus pneumoniae*. The key respiratory pathogens that we want to target in COPD exacerbations when treating patients with relevant risk factors empirically include *Haemophilus influenzae*, *Moraxella catarrhalis*, *Streptococcus pneumoniae*, and *Pseudomonas aeruginosa*. The newer respiratory fluoroquinolones (moxifloxacin and gemifloxacin) were introduced to the market in the years 1999 and 2003, respectively. The MICs of moxifloxacin and gemifloxacin can be compared to the MIC of levofloxacin for these bacterial pathogens (Table 1). Based on the results of these in vitro studies, we see that while all three agents have sufficient coverage for the most common respiratory pathogens, gemifloxacin has superior activity against *Haemophilus influenzae* and *Moraxella catarrhalis* compared to the others. Furthermore, the results demonstrate that the newer agents demonstrate enhanced activity compared to levofloxacin in inhibiting *Streptococcus pneumoniae* and *Pseudomonas aeruginosa* [21].

In summary, gemifloxacin and moxifloxacin demonstrate improved in vitro activity against key respiratory pathogens implicated in COPD exacerbations, and they demonstrate improved pharmacodynamics when compared with levofloxacin, an older respiratory fluoroquinolone. While these two agents have some differences, neither drug has any significant clinical advantage over the other, and both agents serve as useful agents in treating COPD exacerbations associated with bacterial infections. However, there is still concern about the potential for the emergence of resistance if these agents become widely and inappropriately used for the management of respiratory tract infections. The future of fluoroquinolones may depend on how cautiously we utilize these newer agents based on the pathogen we most likely suspect to be the cause of the infection [21]. To date, comparative trials involving respiratory fluoroquinolones are lacking. There is a need for more studies that can inform appropriate antibiotic therapy for COPD exacerbations and will address key concerns including resistance in the context of COPD exacerbation treatment [22].

##### Antivirals

Like bacterial pathogens, respiratory viruses can trigger COPD exacerbations. Viruses that can contribute to exacerbations include rhinovirus, influenza virus, and coronavirus [11]. Studies of respiratory infection in COPD exacerbations are summarized in Table 2.

Approximately 50% of COPD exacerbations are associated with viral infections; the majority of those cases are related to rhinovirus [38]. Therefore, antiviral therapeutic interventions should be considered. While rhinovirus is commonly seen as a viral trigger of COPD exacerbations, studies which tested antivirals targeting rhinovirus in asthmatics did not show significant clinical benefits. However, a study examining neuraminidase inhibitors, the mainstay of therapy for the influenza virus, demonstrated an improvement in reducing hospitalizations in patients with asthma and COPD [39]. SARS-CoV-2, the virus that causes COVID-19, has been associated with COPD exacerbations as well. COPD patients with COVID-19 co-infection who present symptoms that require changes to their maintenance medications meet the criteria for COPD exacerbation [5]. The ACTT-1 trial evaluated the differences between remdesivir and the placebo in the treatment of COVID-19. The results showed improvements in recovery time in patients hospitalized due to coronavirus and COPD or asthma who had lower respiratory symptoms when treated with remdesivir compared with the placebo. Similarly, in the RECOVERY trial, findings demonstrated a reduction in rates of mortality in patients with COVID-19 that required oxygen therapy or mechanical ventilation when treated with dexamethasone compared to the placebo. These results included patients with underlying chronic respiratory disorders [39]. Additionally, nirmatrelvir/ritonavir (Paxlovid) is warranted in patients with SARS-CoV-2 that are immunocompromised or have any conditions that put them at risk of severe symptoms, including COPD or asthma [40]. Further studies are warranted to determine the effects of antivirals on COPD exacerbations, as COPD is thought to be related to an increased susceptibility to viruses due to impairment in immunity and interferon response [39].

#### 3.1.2. Mucoregulators

##### N-acetylcysteine

COPD exacerbations have a complex pathophysiology. Oxidative stress is a significant factor in COPD pathogenesis. N-acetylcysteine (NAC) is an antioxidant and mucolytic that exerts its antioxidant effects by reducing disulfide bonds found in mucus, decreasing mucus viscosity. Based on its mechanism, it is reasonable to assume that this mucolytic may have beneficial effects on patients with COPD. However, the GOLD and NICE guidelines do not recommend using this agent, and there is limited evidence that NAC has any clinical benefit, despite its theoretical advantages. One systematic review and meta-analysis, which included fifteen studies, found that patients that used NAC had improved COPD symptoms and a faster exacerbation-resolution time. The authors found that NAC improved lung function and overall clinical outcomes in patients. The studies that were included in this meta-analysis were small, and larger studies are necessary to evaluate if NAC has a role in the pharmacotherapy of patients experiencing COPD exacerbations [41].

#### 3.1.3. Bronchodilators

##### Beta-2 Agonists

Beta-2 receptors are predominantly found on bronchial smooth muscle. Activation of these receptors causes bronchodilation, enabling patients with COPD to breathe easier. Beta-2 agonists can be short- or long-acting. Short-acting beta-agonists (SABAs) are used primarily during exacerbations due to their ability to relieve symptoms rapidly. Albuterol is the most frequently used SABA since it has the quickest onset of action, relieving symptoms within five minutes. Long-acting beta-agonists (LABAs) are preferred for maintenance therapy in patients with COPD and asthma, as their duration of action is prolonged and lasts throughout the day [42].

##### Muscarinic Antagonists

Muscarinic receptors are found on smooth muscle, including on bronchi, and cause constriction when activated. Muscarinic antagonists, including ipratropium, tiotropium, and glycopyrrolate, antagonize these receptors, resulting in bronchodilation. These agents can be short- or long-acting. As with beta-agonists, short-acting agents (SAMAs) are preferred for treating exacerbations, and long-acting agents (LAMAs) are recommended as maintenance therapy for patients with COPD [43].

Short-acting beta-agonists, with or without short-acting muscarinic antagonists, are recommended as a first-line treatment for patients experiencing COPD exacerbations [5]. Both bronchodilators can be administered via a metered dose inhaler (MDI) or via nebulization. In the past, administering these medications via nebulization was considered more effective than metered dose inhalers. A Cochrane review from 2013 found no difference in the length of hospital stays in adult patients when administered bronchodilators via MDI versus nebulization. However, this review examined patients with asthma and excluded those with COPD [44]. The American College of Chest Physicians/American College of Asthma, Allergy, and Immunology’s evidence-based guidelines and a meta-analysis by Turner et al. both found no differences in efficacy outcomes or adverse effects when using nebulizers versus MDIs when administering bronchodilators. They conclude that the decision regarding which device to use should be made based on availability, cost, and patient and provider preference [45,46]. However, numerous studies have found that nebulizers spread aerosols which can increase the spread of infection. They are also generally more expensive and require maintenance [47,48]. Since the literature has established that both routes of administration have equivalent efficacy, it is reasonable to try to use MDIs with or without a spacer device instead of nebulization. The recommended dosages for bronchodilators differ based on the formulation by which the drug is administered. SABAs should be administered every three to four hours via MDI or every five to six hours via nebulization. SAMAs should be provided every four to six hours regardless of how they are administered [49].

##### Methylxanthines

This class of bronchodilators, which include aminophylline and theophylline, act by inhibiting phosphodiesterase. They can be considered as an alternative therapy in patients with COPD who do not respond to traditional treatments. There is limited evidence supporting their use, and numerous studies found conflicting results in terms of their efficacy and safety. A Cochrane review analyzing data from four randomized clinical trials found inconsistent results regarding the efficacy of methylxanthines, with a significant number of adverse effects and toxicities reported with their use [50]. Another study evaluated the published literature on the benefits of methylxanthines and concluded that their benefits are not well established and that methylxanthines should be avoided due to their adverse effects. However, in patients who do not respond to first-line treatment, intravenous methylxanthines can be considered if administered with careful monitoring [51]. The GOLD and NICE guidelines both do not recommend using methylxanthines in patients with COPD exacerbations [5,52].

##### Future of Bronchodilators in COPD Exacerbations

Bronchodilators are considered a mainstay of treatment for patients with COPD. While these drugs play a central role in maintenance therapy, treating exacerbations, and preventing exacerbations, there has been little advancement in this drug class over the past few decades. Current bronchodilators are limited to beta-agonists, muscarinic antagonists, and xanthines. There are many broncho-dilating drugs in the pipeline, in both preclinical and clinical trials, that aim to identify novel targets and overcome various limitations that are present with existing bronchodilators. While many of the drugs under investigation are studied as maintenance therapy, there is hope that these new agents will prevent recurrent exacerbations and can potentially be used to treat COPD exacerbations as well. Selective phosphodiesterase (PDE) inhibitors, bitter taste receptor (TAS2R) agonists, E prostanoid receptor 4 (EP4) agonists, Rho kinase inhibitors, calcilytics, Peroxisome proliferator-activated receptor-γ (PPAR-γ) agonists, relaxin receptor 1 agonists, soluble guanylyl cyclase (sGC) stimulators, and pepducins are all novel drug classes that are currently being explored for COPD treatment [53,54].

Phosphodiesterase inhibitors are not a new drug class; however, their role in COPD treatment is limited, as xanthines are not recommended for routine use, and roflumilast is only used as a last-line maintenance treatment (not as a treatment for exacerbation therapy). Current studies are investigating other potential phosphodiesterase inhibitors. There are many PDE isoenzymes, each selectively regulating unique cGMP and cAMP subcellar molecules, which can each be potential targets in COPD treatment [53,55]. Drugs with combinations of PDE3 and PDE4, PDE4 and PDE5, and PDE4 and PDE7 have all been studied, with many failing preclinical trials for lack of safety or due to significant adverse-effect profiles [56,57,58,59,60].

Another class of bronchodilators that has recently been identified in the treatment of COPD are agonists of bitter taste receptors [61]. Stimulation of these receptors causes triple the bronchodilation seen with beta-agonists [61]. These receptors are found in multiple areas of the human body; however, the discovery of compounds that can selectively agonize receptors located in respiratory epithelia and smooth muscle has proven to be a challenge [53].

Numerous studies find that the stimulation of prostaglandin E2 receptors, specifically EP4, has bronchodilatory effects [62,63,64]. There are various compounds that have been identified that activate PDE4; however, to date, none have been studied in humans [53].

RhoA (an activator of the Rho kinases, ROCK1, and ROCK2) is a key regulator in smooth muscle and airway constriction. Inhibition of RhoA has been determined to cause bronchodilation, and numerous studies find that in asthmatics and patients with other airway diseases, there is an upregulation of RhoA receptors [53,65,66]. Some inhibitors of these receptors have been approved in countries outside the United States for various indications. However, there have been no Rho Kinase inhibitors with approved indications for airway diseases [67]. Receptors that bind to these drugs are found in the cardiovascular system as well, leading to significant adverse effects and limiting the potential of this drug class [68].

Calcilytics are another class of bronchodilators. These drugs target the G-protein-coupled calcium-sensing receptor. When agonized, these receptors cause bronchoconstriction. Several studies have found the presence of upregulation of these receptors in patients with airway diseases [53]. Calcilytics negatively modulate the G-protein-coupled calcium-sensing receptors, acting locally, without systemic effects on calcium [69]. Inhaled calcilytics are under investigation as a novel drug class for treating COPD [53].

PPAR-γ agonists are a further class of bronchodilators that have recently been studied for their effects on COPD and other airway diseases [70,71,72]. PPAR-γ causes bronchodilation; however, studies found conflicting data on whether PPAR- γ activators cause a considerable enough impact on airway smooth muscle. Preclinical studies have shown airway relaxation with the use of these drugs; however, the results have not been proven to be clinically significant [53].

Relaxin family peptide receptor 1 is found on bronchial smooth muscle and is activated by relaxin-2. Preclinical studies show that the modulation of these receptors offers potential benefits. One drug, serelaxin, demonstrated considerable bronchodilation in numerous studies [73,74,75]. However, it has a short half-life, and modifications of serelaxin are necessary for the practical use of the drug [53]. There are a small number of drugs with this mechanism of action that are currently under investigation in preclinical testing; however, many of the studies are looking for long-acting relaxin agonists for indications other than airway diseases [76,77].

Soluble guanylyl cyclase (sGC) is activated by nitric oxide and causes dilation in the airways, cardiovascular system, and many other parts of the human body. Moreover, sGC stimulators theoretically offer major benefits to patients with COPD; however, there is potential for significant adverse cardiovascular effects [78,79]. Additionally, patients frequently develop a tolerance to nitric oxide compounds; therefore, the development of a drug with this mechanism has proven to be complex [53].

Another drug class, pepducins, has a complex mechanism of action. These drugs, through targeting G-protein-coupled receptors, have the potential to promote bronchodilation. Various studies are currently investigating the role pepducins play in treating airway diseases [80,81].

While there has been little advancement in the way of COPD treatment in the past years, currently, there are many new drugs being studied, each with unique mechanisms of action. There are nine novel bronchodilator classes that have drugs in preclinical and clinical testing, with the promise of treating COPD and reducing rates of morbidity and mortality.

#### 3.1.4. Anti-Inflammatories

##### Corticosteroids

In patients with COPD, there is often increased inflammation found in the lungs, especially during an acute exacerbation; therefore, treatments for COPD commonly include anti-inflammatory drugs. Inhaled corticosteroids are frequently used as maintenance therapy in patients with COPD. Systemic steroids, however, are reserved for patients experiencing moderate-to-severe exacerbations [5]. Numerous studies have found that using systemic glucocorticoids, either oral or intravenous, to manage an exacerbation can decrease rates of relapse and rehospitalization [82,83,84]. Generally, when patients are hospitalized for COPD exacerbations, they receive systemic corticosteroid therapy intravenously. Oral therapies are significantly cheaper than intravenous medications. However, not all oral medications reach the adequate systemic concentrations necessary for achieving a therapeutic response. The literature has shown that using oral corticosteroids in COPD patients resulted in similar clinical outcomes compared with intravenous therapy. In one clinical trial by de Jong et al., oral therapy was compared with intravenous therapy in patients with COPD exacerbations. The results between the two treatment groups were similar, indicating that systemic therapy does not need to be administered solely via the intravenous route. Oral therapy can also be considered if tolerated by patients [38]. It was considered standard therapy to use systemic corticosteroids for a prolonged period in COPD exacerbations, with a typical duration of ten to fourteen days. Prolonged exposure to corticosteroids is associated with significant adverse effects including hyperglycemia, osteoporosis, and risk of infection [85]. In 2013, the REDUCE trial evaluated the safety and efficacy of short-term oral corticosteroids compared with the standard of care at the time. The results of the study found that using a five-to-seven-day duration of therapy resulted in similar outcomes and fewer adverse effects compared with a fourteen-day duration of steroid therapy [86]. A review of the additional literature found similar results from numerous other studies. These studies demonstrated that using a shorter duration of steroid therapy led to fewer adverse effects, with similar efficacy compared with extended durations of therapy [87]. The GOLD guidelines updated their recommendations after the publication of the REDUCE trial, and now recommend therapy for five to seven days [5]. Dosing of corticosteroids is based on equivalents of prednisone. The GOLD guidelines recommend using equivalents of forty milligrams of prednisone per day for five days, while the NICE guidelines from the United Kingdom recommend thirty milligrams of prednisone for seven to fourteen days [5,52]. There is insufficient literature on which dose is more effective, with inconsistent results from the few studies that have been published. Often, in clinical practice, the dose of the steroid used is higher than what is recommended by guidelines. One randomized, open-label trial evaluated the difference between using a fixed dose of forty milligrams of prednisone equivalents versus a patient-specific dose. The personalized dose was based on five factors that helped determine the severity of the COPD exacerbation that the patient experienced. The results of the trial found that using patient-specific factors to determine the dose of corticosteroid resulted in higher dosing and better patient outcomes. The average dose of corticosteroid was more than sixty milligrams of prednisone equivalents in the patient-specific group. The results of this trial indicate that despite the recommendations made by the GOLD and NICE guidelines, higher corticosteroid dosing can be considered based on patient-specific factors and the severity of COPD exacerbation [88]. The benefits of corticosteroids are largely seen in patients with mild and moderate exacerbations. There is limited evidence demonstrating the benefits of corticosteroids in patients admitted to the intensive care unit due to severe COPD exacerbations [89]. The studies that looked at patients in the ICU had contradictory results, with some studies finding no benefit when using corticosteroids in patients with COPD exacerbations in the ICU, while a few papers found that corticosteroids improved patient outcomes [90].

There is evidence that indicates that there is a correlation between elevated eosinophil count and the severity of COPD exacerbation [91]. However, there is little data supporting the use of eosinophil count as a biomarker to determine whether corticosteroids should be used in patients experiencing a COPD exacerbation. One randomized, placebo-controlled trial evaluated the effects of using biomarker-guided therapy in patients being treated for COPD exacerbations. In the investigative arm, clinicians used eosinophil count to determine whether to add corticosteroids to patients’ treatment regimens. In the standard-of-care arm, all patients received corticosteroids. The authors found that using an eosinophil count to guide therapy enabled around 50% of patients to avoid exposure to corticosteroids and ultimately have better outcomes. The trial was small and had many limitations, and further studies are needed to evaluate whether using eosinophil count to guide therapy has a place in clinical practice [92].

##### Corticosteroids in COPD Patients with or without COVID-19

During the early stages of the COVID-19 pandemic, treatment recommendations were based on low-quality evidence given that there was limited data available. As the pandemic progressed, and enormous amounts of research were produced daily, treatment recommendations evolved. Initially, corticosteroid use was not recommended for patients with COVID-19 due to concerns regarding delayed clearance of the virus [93]. Clinicians were unsure how to treat patients with comorbid conditions that warranted corticosteroid use. The tremendous benefit of corticosteroids in COPD is well established; however, it was presumed that COPD patients were at elevated risk of contracting severe acute respiratory syndrome coronavirus 2 (SARS-CoV-2), and a risk-benefit analysis was necessary to determine optimal therapy management. Clinicians questioned whether to treat COPD patients in the presence or absence of COVID-19 with inhaled corticosteroids for maintenance therapy and systemic corticosteroids for exacerbation treatment [94,95].

Corticosteroids are immunosuppressants, and numerous studies have found that there is an increased risk of contracting an infection and developing viral pneumonia in those that take both inhaled and systemic corticosteroids [96]. In 2020, during the first few months of the pandemic, the World Health Organization issued a warning against using corticosteroids in COVID-19 treatment due to the potential for delayed clearance of the virus with steroid use [93]. This warning triggered concern in clinicians and patients that corticosteroid use can perhaps increase the risk of contracting COVID-19, and many patients were reluctant to continue maintenance inhaled corticosteroid therapy for COPD [94]. Studies analyzing the correlation between comorbidities and COVID-19 incidence found conflicting data. Some of the literature observed no difference in the number of COVID-19 cases in patients with COPD compared to those without; however, some studies found that cases of COVID-19 were indeed greater among patients with COPD [97,98,99,100]. Due to insufficient evidence at the time, the GOLD guidelines, NICE guidelines, and numerous others recommended that patients with COPD without COVID-19 should not discontinue their maintenance inhaled corticosteroid therapy, and patients with COPD exacerbations should be treated with systemic steroid therapy as appropriate [101,102].

Recommendations for COPD therapy in patients without COVID-19 were determined based on insufficient evidence advocating against the use of standard treatment. In patients that contracted COVID-19, recommendations regarding care were more complicated to determine. Patients with COPD and COVID-19 are at higher risk of developing a COPD exacerbation, and appropriate management of their exacerbation is imperative. During the initial few months of the pandemic, there were numerous small studies that found that the use of corticosteroids in COPD exacerbations in the presence of COVID-19 did not worsen patient outcomes despite what was theorized by many [103]. Additionally, some literature demonstrated that using inhaled corticosteroids might prove beneficial and decrease the risk of contracting COVID-19 [104]. Therefore, despite WHO’s recommendation against corticosteroid use in COVID-19 patients, the results of these studies concluded that systemic corticosteroids should be used, albeit cautiously, to treat COPD exacerbations in patients that have concomitant COVID-19 infections [5].

Recommendations regarding corticosteroid use in COVID-19 changed with the publication of the RECOVERY trial (a large, randomized, open-label trial, which published preliminary findings in January 2020). RECOVERY produced strong evidence supporting the use of corticosteroids to treat COVID-19. This study, in addition to other studies that took place around the same time, found that using systemic corticosteroids reduced mortality rates in various patient populations [105]. WHO withdrew its recommendation against the use of corticosteroids after the results of these trials became published. Subsequently, numerous guidelines globally updated their recommendations in support of the use of corticosteroids in the treatment of COVID-19 in certain patient populations [105]. Since the update in recommendations, there has been no concern over the use of corticosteroids in patients with COPD with or without COVID-19. Normal steroid therapy should be used in patients with COPD regardless of whether the patient is at risk of contracting COVID-19 or tests positive for the disease. Patients should continue using inhaled corticosteroids for maintenance therapy without concern, and treatment of exacerbations should include systemic corticosteroids as appropriate based on standard recommendations [105]. To further support the updated recommendations, a recent Cochrane review from 2022 found no difference in mortality and a potential slight benefit in those that use inhaled corticosteroids with confirmed COVID-19. The quality of evidence is low; however, the results clearly indicate that no harm was seen in patients that used inhaled steroids with COVID-19 [105].

#### 3.1.5. Long-Term Oxygen Therapy and Beta-Blocker Therapy in COPD Exacerbations

Hypoxia frequently accompanies COPD exacerbations due to inadequate oxygenation in patients. Prolonged hypoxia and subsequent hypoxemia are associated with significant reversible and irreversible damage [106]. Consequently, oxygen therapy is a critical element in COPD exacerbation management. Oxygen therapy refers to the administration of oxygen at concentrations greater than those found in room air and is used when patients are unable to maintain adequate oxygen saturation while respirating on their own [107]. Determination of optimal oxygen dosing in COPD exacerbations is necessary, and numerous studies investigate the differences in using standard high-flow oxygen versus titrating oxygen based on target blood saturation (abbreviated spO2).

A randomized, controlled, parallel-group trial investigated the mortality benefits of using titration-based dosing for oxygen therapy in acute COPD exacerbations compared to standard high-flow oxygen. The results showed that targeting an spO2 between 8 and 2% and titrating oxygen doses based on these levels decreased mortality, hypercapnia, and respiratory acidosis compared to those that received high-flow oxygen only [108]. Another study found that targeting 90–92% oxygen saturation was optimal, as higher oxygen levels resulted in an increased incidence of hypercapnia with no added benefit to patients [109]. The results of a recent study, published in 2020, correspond to the findings of these previous trials. The authors found a statistically significant decrease in mortality rates in patients with titrated oxygen targeting an oxygen saturation of 88–92% compared to patients administered doses based on higher spO2 targets [110]. Based on the data from available literature, the GOLD guidelines recommend that the dosing of oxygen should be titrated on an individual basis with a target oxygen saturation of 88–92% [5]. Numerous studies support the recommendation that the correction of hypoxemia should be conducted even if hypercapnia occurs as long as the oxygen saturation remains within 88–92% [109,111]-YR-3. Once patients reach target spO2 and are stable at their dose of oxygen, the duration of their oxygen therapy must be defined.

Long-term oxygen therapy, commonly referred to as LTOT, refers to the prolonged use of oxygen in the presence of stable COPD in the outpatient setting. The benefits seen with LTOT are significant, with numerous, large-scale studies conducted that support its use. However, excess oxygen therapy can lead to significant adverse effects if it is not used as indicated. A summary of the trials supporting LTOT can be found on UpToDate [112]. A review of the literature evaluating the benefits of LTOT in stable COPD is out of the scope of this article; however, a basic understanding of LTOT is necessary, as many patients experiencing acute exacerbations are initiated on continuous oxygen at the time of their discharge from the hospital. LTOT has been proven to be beneficial in patients with severe, persistent hypoxia only when initiated in stable patients. Specific eligibility criteria are required for patients to be initiated on LTOT [113].

After the resolution of an acute COPD exacerbation, patients are often discharged while still hypoxic, as hypoxia can persist for a few months post exacerbation. Thus, these patients necessitate oxygen therapy in the outpatient setting [114]. Many studies find that the majority of these patients experience the resolution of their hypoxia within 30–60 days and reevaluation of their outpatient oxygen use is necessary at that time [115,116,117]. Upon reevaluation of oxygen needs, oxygen should only be continued if patients remain eligible based on LTOT criteria [113]. Inappropriate continuation of oxygen therapy in this patient population can lead to unnecessary oxygen use and potential harm. One study found that up to 60% of patients that were initiated on LTOT at the time of acute exacerbation would have been eligible for discontinuation of their oxygen had they been reevaluated at the appropriate follow-up time [117]. Therefore, if a patient is discharged with oxygen after an exacerbation, it is critical that a reassessment of their hypoxia is conducted after one to two months to ensure that their continuous outpatient oxygen use is short-term and not unnecessarily prolonged [117].

The GOLD guidelines suggest avoiding the use of beta-blockers for the treatment of COPD exacerbations in patients that do not have any known cardiovascular co-morbidities that would otherwise warrant their use [118]. Previously, there were several observational studies that were conducted to determine whether beta-blockers played a role in treating COPD exacerbations; patients included in these observational studies had COPD as well as a clear cardiovascular indication that required beta-blocker therapy. This was an important area that needed investigation as it was found that many patients with CVD and COPD were being under-treated due to a hesitance to prescribe beta-blockers in patients with COPD for fear of worsening lung function [119]. A systematic review conducted by Gulea et al. investigated the impact of beta-blocker therapy in patients with COPD using data from 23 observational studies and 14 Randomized Controlled Trials. A pooled analysis of the results of the 23 observational studies demonstrated that in patients with COPD that received beta-blockers, there was a 23% lower risk of COPD exacerbations compared to those that did not receive beta-blockers [120]. However, a reduction in FEV1 was seen in patients receiving propranolol compared to those that received a placebo. This reduction in FEV1 was only seen with propranolol, a non-selective beta-blocker [120]. These findings are consistent with findings from a meta-analysis conducted by Du et al. which included 15 observational cohort studies. The results of this analysis demonstrated a greater risk reduction of 37% in COPD acute exacerbations in patients with COPD receiving beta-blockers. In addition, there was a 28% relative risk reduction in all-cause mortality in those patients receiving beta-blockers [121]. In summary, observational cohort studies demonstrate that cardio-selective beta-blockers were well tolerated and were effective in minimizing exacerbations and overall death in patients with COPD and underlying cardiovascular diseases. Although there were several observational studies to support the use of beta-blockers in patients with COPD and underlying cardiovascular diseases, a randomized controlled trial was warranted to determine whether patients with COPD and no cardiovascular diseases would benefit from therapy with beta-blockers. In the BLOCK COPD Trial, patients with COPD were randomly assigned to receive either metoprolol or a placebo. The goal was to determine whether there would be a one-year risk reduction in all-cause mortality, exacerbations of COPD, or cardiovascular events in patients receiving metoprolol [122]. Key exclusion criteria were the absence of an indication for a beta-blocker which was defined as a coronary revascularization within the past 3 years or congestive heart failure with a left ventricular ejection fraction of less than 40%. The results demonstrated that there was no significant difference in time until the first exacerbation or rates of exacerbations between the two groups. However, in those patients that received treatment with metoprolol, there was evidence pointing towards an increase in severe and very severe exacerbations, defined as those exacerbations that led to hospitalization and those that led to intubation or mechanical ventilation, respectively. There were eleven deaths in the metoprolol group compared to five deaths in the placebo group; however, this finding was not statistically significant. There was an increase in all-cause hospitalization, an increase in nonfatal adverse events, and an increase in nonfatal serious COPD exacerbations in the metoprolol group compared to the placebo group. Regarding changes in FEV1, six-minute walk distance, and St. George’s Respiratory Questionnaire score, there was no significant difference between the two groups; however, patients in the metoprolol group did experience worsening symptoms such as shortness-of-breath. During the second interim analysis period, the trial was cut short due to the increase in severe exacerbations, very severe exacerbations, and a trend towards increased mortality seen in the metoprolol group [122]. While the use of beta-blockers is generally well tolerated and effective in patients with an evidence-based cardiovascular indication for beta-blockers and underlying COPD, the results of the BLOCK COPD Trial indicate that they should not be used in patients who do not have underlying CVD as beta-blockers can worsen health status, increase the risk of severe exacerbations leading to hospitalization or ventilation, and contribute to an increase in unfavorable symptoms. These findings are consistent with the recommendation of the GOLD guidelines and support reserving the use of beta-blockers for patients with COPD and overt CVD. Prescribers should not deter from using cardio-selective beta-blockers when warranted in these patients.

#### 3.1.6. Vitamin D

Vitamin D is an essential nutrient and is involved in numerous functions in the human body. Vitamin D has antimicrobial effects by inducing antiviral and antibacterial effector mechanisms. It is also associated with modulating immune responses, and there is strong evidence to indicate that low levels of serum 25-hydroxyvitamin D, the active form of vitamin D, are correlated with poor immune health. Recent literature has shown that vitamin D levels are often lower in patients with COPD compared to levels in healthy individuals [123]. Newer studies are now investigating the role that vitamin D plays in preventing and treating COPD exacerbations [124,125]. Data evaluating vitamin D’s role in preventing COPD are controversial. Some of the literature indicates that long-term vitamin D supplementation prevents exacerbations, and some studies find no correlation [125]. One meta-analysis reviewing four clinical trials found that using vitamin D in patients with COPD only has benefits if their baseline serum 25-hydroxyvitamin D is chronically low [125]. Most studies investigated the benefits of vitamin D in preventing exacerbations, while there are few published studies on its effects in treating exacerbations. Despite this, the GOLD guidelines recommend checking vitamin D levels in patients hospitalized with severe COPD exacerbations and repleting them if the levels are below ten nanograms per milliliter (normal levels are between twenty and forty nanograms per milliliter) [5].

#### 3.1.7. Personalized Medicine, Epigenetics and COPD

Epigenetics is defined as the study of heritable changes in gene expression that occur without alteration in the DNA sequence. Epigenetic marks can be segmented into three classes: DNA methylation, post-translational histone modifications, and non-coding RNAs. The three classes are induced by environment, diet, disease, and aging [126]. Epigenetics provides the connection between genetic factors and environmental conditions [127]. Epigenetic mechanisms are involved in the regulation of gene expression in chronic lung diseases including asthma and COPD.

DNA methylation has been shown to have a fundamental role in the presence and development of COPD. In epithelial cells and alveolar macrophages from COPD patients, DNA methylation of the promoter regions of proinflammatory genes has been observed [128]. DNA methylation can be influenced by cigarette smoking [129]. In addition, epigenetic changes have been reported in promoter regions when exposed to air pollution [129].

To improve the treatment of COPD, novel approaches using a personalized approach are warranted [130]. Such therapeutic approaches could arise from epigenetic studies. Epigenetic marks are attractive for targeted therapies. It is indeed an aspiration that the development of epigenetic technologies will result in effective clinical therapies for COPD.

## 4. Conclusions

COPD is a leading global cause of death. It is an irreversible, inflammatory airway disease predominantly caused by prolonged exposure to toxins. Exacerbations of COPD are frequently precipitated by bacterial and viral infections. Significant progress has been made in the treatment of COPD with diverse anti-microbials, mucoregulators, and the use of bronchodilators and anti-inflammatories. As noted in this review, with advances made in personalized medicine, there is a need to explore new therapeutic approaches including epigenetic modifications. Additionally, modulation of the oral and lung microbiomes by replacement of pathogens with commensal microorganisms should be investigated as a therapeutic path. Future work will hopefully elucidate whether personalized medicine can contribute to effective COPD pharmacotherapy.

## Figures and Tables

**Table 1:** Antibiotic Minimum Inhibitory Concentration (MIC).

	MIC_90_ of Indicated Drug (μg/mL)		
**Bacterial Organism:**	Gemifloxacin	Moxifloxacin	Levofloxacin
*Haemophilus influenzae*	0.03	0.06	0.06
*Moraxella catarrhalis*	0.015	0.03	0.03
*Streptococcus pneumoniae*	0.03	0.12	1.0
*Pseudomonas aeruginosa*	>8.0	8	32

Data derived from [21].

**Table 2:** Viral infections detected via PCR in patients experiencing COPD exacerbations.

Study Author	Viral Infection Detected	Detection Technique	Comments
Ruiz-González et al. [23]	Influenza A (*n* = 34; 39.5%) Rhinovirus (*n* = 20; 23.3%) Coronavirus (*n* = 10; 11.6%) Respiratory syncytial virus (*n* = 9; 10.5%)	RT-PCR	Out of 127 patients included in the study, 57 patients (44.9%) had a viral infection detected via PCR, and 29 patients (22.8%) had both bacterial and viral infections detected via PCR. The four most prevalent viral isolates are listed.
Kim et al. [24]	Influenza virus (*n* = 34; 14.1%): Influenza A (*n* = 33; 13.7%) Influenza B (*n* = 1; 0.4%) Rhinovirus (*n* = 25; 10.4%) Parainfluenza (*n* = 23; 9.5%) Human coronavirus (*n* = 15; 6.2%)	RT-PCR	Notably, 101 (41.9%) of the included patients with acute COPD exacerbations had respiratory viral infections detected. The four most prevalent isolates are listed. [Note: this study was published in 2016, before the identification of SARS-CoV-2, COVID-19]
Vanspauwen et al. [25]	Zero cases of mimivirus detected	PCR	The presence of mimivirus antibodies in patients with pneumonia suggests a possibility that this virus is a respiratory pathogen and may potentially play a role in respiratory infections. PCR tests performed on the sputum samples of 220 patients with stable COPD, and those experiencing acute exacerbation indicate that this virus does not play a role in COPD as none of the PCR tests detected cases of mimivirus.
Perotin et al. [26]	Human rhinovirus (*n* = 9; 20%) human metapneumovirus (*n* = 8; 18%) Influenza A (*n* = 2; 4%) Influenza B (*n* = 1; 2%)	Multiplex PCR	Of the 45 patients included in this study, 20 patients (44%) had a viral respiratory infection associated with their AECOPD. The four most prevalent isolates are listed.
Chen et al. [27]	Influenza-positive cases (*n* = 90) Influenza A (*n* = 68) Influenza B (*n* = 22)	PCR	PCR only tested for influenza, and 925 patients were included in the study.
Biancardi et al. [28]	In hospitalized patients: Influenza A (31%) Rhinovirus (27%) Respiratory syncytial virus A/B (10%) Non-hospitalized patients: Influenza A (*n* = 642; 31%) Rhinovirus (*n* = 565; 27%) RSV A/B (*n* = 209; 10%)	Multiplex PCR	In 102 patients hospitalized for COPD exacerbation, 59 patients (58%) had a respiratory viral infection. The four most prevalent isolates are listed. Out of 8811 non-hospitalized patients experiencing COPD exacerbation, 5599 of those patients (64%) had viral respiratory pathogens identified via PCR. The four most prevalent isolates are listed.
Kan-O et al. [29]	hMPV (*n* = 7; 15.9%) Parainfluenza virus (*n* = 4; 9.1%) HRV/enterovirus (*n* = 2; 4.5%) coronavirus (*n* = 2; 4.5%) respiratory syncytial virus (*n* = 2; 4.5%)	Multiplex PCR	In patients experiencing acute COPD exacerbations, 17 of those patients (38.6%) had respiratory viral infections identified via PCR.
Yormaz et al. [30]	Rhinovirus (25%) Influenza A (13.1%) Coronavirus (11.8%)	PCR	In a study that included 110 patients, 50 of those patients (45.5%) had respiratory viral infections identified via PCR.
Koul et al. [31]	Influenza (*n* = 18; 7.7%) Rhinovirus (*n* = 11; 4.7%) RSV-A (*n* = 5; 2.1%) Parainfluenza virus (*n* = 4; 1.7%)	PCR	In a study conducted in India, which included 233 patients, 46 of those patients (19.7%) had respiratory viral infections identified via PCR.
McManus et al. [32]	Rhinovirus (*n* = 32) Adenovirus (*n* = 10) Parainfluenza-3 (*n* = 5) Influenza A-H3 (*n* = 3)	Multiplexed, nested PCR	Of the 136 patients included in this study, 37% had respiratory viral infections identified via PCR.
Yin et al. [33]	Influenza A (9.5%) Human rhinovirus (8%) Influenza B (5.7%)	RT-PCR	A total of 264 patients were included in a study that was conducted in Shanghai, and 72 of those patients (27.3%) had respiratory viral infections identified via PCR.
Van Rijn et al. [34]	Rhinovirus (*n* = 14; 61%) Influenza A (*n* = 3; 13%) coronavirus NL63 (*n* = 2; 9%) Coronavirus OC43 (*n* = 1; 4%) Parainfluenza virus 3 (*n* = 2; 9%) Parainfluenza virus 4 (*n* = 1; 4%)	qPCR	A total of 88 patients from the Bergen COPD exacerbation study were included, and 23 of those patients (26%) had viral respiratory infections identified via qPCR.
Camargo et al. [35]	Respiratory syncytial virus (8%) Rhinovirus (4%) Influenza A (3%) Human metapneumovirus (3%)	PCR	Out of 76 patients included in this study, 19 patients (25%) had respiratory viral infections identified via PCR. The four most prevalent isolates are listed.
Beckham et al. [36]	Picornavirus (*n* = 22) Coronavirus 229E/OC43 (*n* = 10) Influenza A/B (*n* = 3) Parainfluenza virus types 1–3 (*n* = 3)	RT-PCR	Out of the 96 patients included, 35 patients had a respiratory viral infection identified via PCR. The four most prevalent isolates are listed.
Ko et al. [37]	Influenza A (7.3%) Coronavirus OC43 (4.6%) Rhinovirus (3.1%) Influenza B (2.7%) Respiratory syncytial virus (2.3%)	PCR	A total of 196 patients were included in this study, which was conducted in Hong Kong; 58 of those patients (22.1%) yielded positive viral PCR results.

## Data Availability

References and databases have been provided.
